# Effective Treatment Recommendations for Type 2 Diabetes Management Using Reinforcement Learning: Treatment Recommendation Model Development and Validation

**DOI:** 10.2196/27858

**Published:** 2021-07-22

**Authors:** Xingzhi Sun, Yong Mong Bee, Shao Wei Lam, Zhuo Liu, Wei Zhao, Sing Yi Chia, Hanis Abdul Kadir, Jun Tian Wu, Boon Yew Ang, Nan Liu, Zuo Lei, Zhuoyang Xu, Tingting Zhao, Gang Hu, Guotong Xie

**Affiliations:** 1 Ping An Healthcare Technology Beijing China; 2 Department of Endocrinology Singapore General Hospital Singapore Singapore; 3 SingHealth Duke-NUS Diabetes Centre Singapore Health Services Singapore Singapore; 4 Health Services Research Centre Singapore Health Services Singapore Singapore; 5 Health Services and Systems Research Duke-NUS Medical School Singapore Singapore; 6 Health Services Research Unit Singapore General Hospital Singapore Singapore; 7 Ping An Healthcare and Technology Co, Ltd Shanghai China; 8 Ping An International Smart City Technology Co, Ltd Shenzhen China

**Keywords:** type 2 diabetes, reinforcement learning, model concordance, short-term outcome, long-term outcome

## Abstract

**Background:**

Type 2 diabetes mellitus (T2DM) and its related complications represent a growing economic burden for many countries and health systems. Diabetes complications can be prevented through better disease control, but there is a large gap between the recommended treatment and the treatment that patients actually receive. The treatment of T2DM can be challenging because of different comprehensive therapeutic targets and individual variability of the patients, leading to the need for precise, personalized treatment.

**Objective:**

The aim of this study was to develop treatment recommendation models for T2DM based on deep reinforcement learning. A retrospective analysis was then performed to evaluate the reliability and effectiveness of the models.

**Methods:**

The data used in our study were collected from the Singapore Health Services Diabetes Registry, encompassing 189,520 patients with T2DM, including 6,407,958 outpatient visits from 2013 to 2018. The treatment recommendation model was built based on 80% of the dataset and its effectiveness was evaluated with the remaining 20% of data. Three treatment recommendation models were developed for antiglycemic, antihypertensive, and lipid-lowering treatments by combining a knowledge-driven model and a data-driven model. The knowledge-driven model, based on clinical guidelines and expert experiences, was first applied to select the candidate medications. The data-driven model, based on deep reinforcement learning, was used to rank the candidates according to the expected clinical outcomes. To evaluate the models, short-term outcomes were compared between the model-concordant treatments and the model-nonconcordant treatments with confounder adjustment by stratification, propensity score weighting, and multivariate regression. For long-term outcomes, model-concordant rates were included as independent variables to evaluate if the combined antiglycemic, antihypertensive, and lipid-lowering treatments had a positive impact on reduction of long-term complication occurrence or death at the patient level via multivariate logistic regression.

**Results:**

The test data consisted of 36,993 patients for evaluating the effectiveness of the three treatment recommendation models. In 43.3% of patient visits, the antiglycemic medications recommended by the model were concordant with the actual prescriptions of the physicians. The concordant rates for antihypertensive medications and lipid-lowering medications were 51.3% and 58.9%, respectively. The evaluation results also showed that model-concordant treatments were associated with better glycemic control (odds ratio [OR] 1.73, 95% CI 1.69-1.76), blood pressure control (OR 1.26, 95% CI, 1.23-1.29), and blood lipids control (OR 1.28, 95% CI 1.22-1.35). We also found that patients with more model-concordant treatments were associated with a lower risk of diabetes complications (including 3 macrovascular and 2 microvascular complications) and death, suggesting that the models have the potential of achieving better outcomes in the long term.

**Conclusions:**

Comprehensive management by combining knowledge-driven and data-driven models has good potential to help physicians improve the clinical outcomes of patients with T2DM; achieving good control on blood glucose, blood pressure, and blood lipids; and reducing the risk of diabetes complications in the long term.

## Introduction

Type 2 diabetes mellitus (T2DM) is a worldwide chronic disease characterized by higher than optimal blood glucose levels. T2DM can lead to multiple complications and increase the risk of death. According to the global report on diabetes of the World Health Organization [1], 3.7 million people died of diabetes in 2012 and the prevalence has been increasing in the past three decades. T2DM and its related complications represent a growing economic burden for many countries and health systems [2]. Diabetes complications can be prevented through better disease control; however, there is still a large gap between the recommended treatment and the treatment that patients actually receive [3].

The treatment of T2DM can be challenging because of the different therapeutic targets and individual variability of the patients, leading to the need for precise, personalized treatment [4]. In addition, patients with diabetes require a sequence of treatments due to chronicity of the condition, each of which may affect the patients’ clinical outcome in the long term. The decision-making for determining a sequence of treatments can be more complex because (1) the impact of a single treatment may not be immediately reflected, and (2) if we regard all of the treatments a patient received chronically as a treatment program, the number of options for the treatment programs is extremely large and finding the best program for an individual patient is a great challenge.

With the explosive increase of electronic medical records (EMRs) and the rapid development of artificial intelligence technology, it has now become possible to teach a model that enables personalized treatment with the best expected clinical outcomes. The treatment of chronic diseases such as T2DM is a sequential decision-making process. Our goal is to develop effective treatment regimens that can dynamically adapt to the varying clinical states and maximize the long-term benefits of patients. Reinforcement learning (RL) [5] is an approach that learns the best policy toward a predefined long-term goal via trial and error to address a sequential decision-making problem. The RL approach has intrinsic advantages of tackling the treatment recommendation problem for chronic diseases. First, by considering the accumulative rewards as the optimization goal, the long-term effect of current decision-making is taken into account. Second, the design of RL leverages all samples in model development by reinforcing actions with a good reward and punishing others with a bad reward. With theoretical and technical developments in recent years, the RL approach has been successfully applied in the health care domain, including for chronic disease management [6-10], critical care [11-14], and other forms of health management [15,16].

The treatment of chronic diseases consists of a sequence of medications or procedures that are determined based on the changing clinical conditions of a patient and the effects from the previous treatment. Tseng et al [6] recently proposed an RL-based model to automate adaptive radiotherapy decision-making for patients with nonsmall cell lung cancer, where the deep Q network (DQN) was used to learn dose decisions based on real clinical data and the synthesized data created by generative adversarial networks [17]. The framework was evaluated in a dataset of 114 patients. The learned dose strategies by the DQN could achieve similar results to those decided by clinicians, yielding feasible and promising solutions for automatic treatment designs. Once a treatment recommendation model is developed, it is imperative to carefully evaluate its validity and effectiveness before wide application. In the clinical domain, a randomized controlled trial (RCT) is often performed to test the efficacy of an intervention. However, RCTs can be costly, unpractical, and infeasible in some clinical scenarios [18]. With the increase of EMR use, a retrospective study has become a reasonable alternative to evaluate models via statistical tests and other data analytics methods.

There is an emerging trend in the literature for effectiveness evaluation on the treatment of chronic diseases [19-21], such as the comparative effectiveness of more or less aggressive treatment intensification strategies in adults with T2DM [19]. In these studies, two types of treatments are compared in terms of a short-term clinical outcome such as the key indicator of the disease and a long-term outcome such as the occurrences of complications or death. When making a comparison in such observational studies, it is crucial to eliminate the influence of confounding factors. For short-term clinical outcomes, multiple logistic regression and the propensity score (PS) method are conventional approaches to adjust the confounders between treatment groups [22]. With respect to long-term outcomes, survival analysis via the Cox proportional hazard model can be applied to adjust the time-invariant or time-varying covariates for two treatment groups [23]. If the covariates change over time and are affected by the previous treatment, the use of marginal structural models [24-26] was proposed to control the confounders. In relation to diabetes treatment, Chen et al [27] assessed the association between treatment concordance with clinical guidelines and related clinical outcomes in patients with T2DM by comparing guideline-concordant and guideline-nonconcordant cohorts. This work is closely related to treatment model evaluation as the treatment groups to be compared are defined based on a given guideline, which can be regarded as a special treatment model that has been verified and commonly accepted. Chen et al [27] considered hospital admission and severe hypoglycemic events as the clinical outcomes of interest. Logistic regressions were used to examine factors associated with the likelihood of having at least one hospital admission and Cox proportional hazard regressions were used to model time to hypoglycemic events.

In this work, we developed treatment recommendation models based on the deep RL approach and then performed a retrospective study to evaluate the reliability and effectiveness of the models. The anonymized data used in our study are derived from the Singapore Health Services (SingHealth) Diabetes Registry [28], which is built based on the EMRs from SingHealth, the largest health cluster in Singapore with 4 hospitals, 5 national centers, 8 polyclinics (primary care clinics), and 3 intermediate long-term-care community hospitals. For treatment recommendation, we successfully applied the deep RL technique in the context of personalized treatment for patients with T2DM, with careful design and formulation for this challenging problem. We built a model that can be used to recommend the medications for patients with T2DM based on their clinical information, including demographic data, vital signs, laboratory tests, disease history, and current medications. Three models were developed for antiglycemic, antihypertensive, and lipid-lowering treatments to enable the comprehensive management of patients with T2DM. We evaluated the effectiveness of our treatment recommendation models by systematically performing a retrospective study on the EMRs of patients with diabetes.

## Methods

### Patient Characteristics

This retrospective study was based on anonymized data of 189,520 patients with T2DM from SingHealth Diabetes Registry between January 2013 and December 2018. The study was approved by the SingHealth Centralized Institutional Review Board with a waiver of informed consent granted. The board deemed that further ethical deliberation was not required as the study involves analysis of an anonymized dataset. All methods performed in this study were in accordance with the relevant guidelines and regulations. The dataset was split into training data (80% with 152,527 patients) for treatment recommendation models, including three types of treatments (antiglycemic, antihypertensive, and lipid-lowing treatment), and test data (20% of data with 36,993 patients) for evaluating the effectiveness of the three treatment recommendation models.

The EMR data for each patient included demographic information, medical history, physical measurements, laboratory data, and physicians’ prescriptions. Demographic information included age, gender, ethnicity, smoking, and others. Medical history included comorbidities, vascular complications, hospital admissions, emergency department visits, and outpatient visits. Physical measurements included systolic blood pressure (SBP), diastolic blood pressure (DBP), heart rate, weight, height, and BMI. Laboratory data included glycated hemoglobin A_1c_ (HbA_1c_), low-density lipoprotein cholesterol (LDL-c), and fasting plasma glucose. For physicians’ prescriptions, we considered only antiglycemic, antihypertensive, and lipid-lowering drugs and their dosages.

As is the case for all EMR data, our dataset contains errors and missing data. The rate of missingness was generally low, with higher rates for variables under the categories of physical measurements and laboratory data. We handled the errors and missing data using the following strategy. During preprocessing, errors were treated as missing values. For missing physical measurements and laboratory data, we substituted the missing data with the value from the closest preceding data point of the same patient within a 1-year time frame. If data were still missing, we proceeded to impute the missing data using the median of the observed values for that variable for all patients without missing data.

### Clinical Outcomes

Two types of clinical outcomes were analyzed: short-term and long-term outcomes. Short-term outcomes were evaluated at the patient-visit level, including blood glucose control, blood pressure control, blood lipids control, and hypoglycemia-related admissions. For the long-term outcomes, we evaluated the occurrences of 5 diabetes complications and death in up to 6 years at the patient level, including myocardial infarction, heart failure, stroke (including ischemic and hemorrhagic strokes), diabetic nephropathy, other microvascular complications (diabetic neuropathy, diabetic eye complications, diabetic foot/peripheral angiopathy), and death.

### Treatment Recommendation Models

The treatment recommendation models were based on the patient’s clinical information from visits as input to recommend three types of treatments as output: antiglycemic, antihypertensive, and lipid-lowering medications. The input clinical information of a patient contains demographic information, lab data, physical measurements, medical history, and prescriptions currently in use. We utilized three models to recommend the three types of medications, and then combined the output of the three models into a comprehensive treatment recommendation.

**Figure 1:**
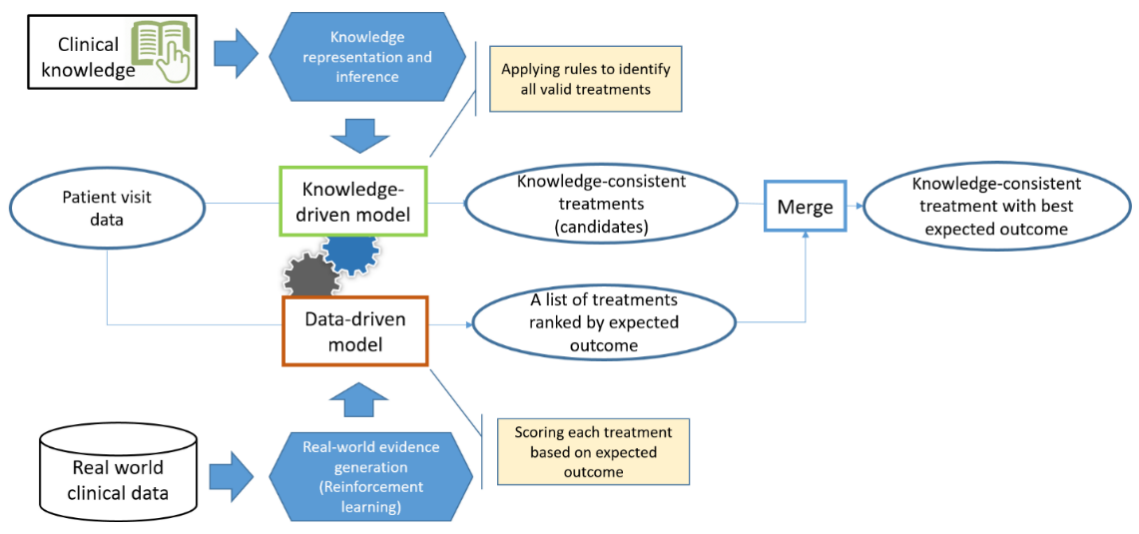
Treatment recommendation model of the “Knowledge + Data” two-wheel-drive method.

Figure 1 illustrates the treatment recommendation approach by combining a knowledge-driven model and a data-driven model. The knowledge-driven model was developed based on the clinical guidelines and expert experiences on managing T2DM [29-34]. For the data-driven model, RL was used to learn the policy of treatment recommendation from real-world data that optimizes a predefined long-term goal via trial and error [35-37]. When integrating these two types of models, the knowledge-driven model was first applied to select the candidate medications, and the data-driven model was used to rank the candidates by the expected clinical outcomes.

### RL-Based Framework of the Data-Driven Model

We trained the RL model on a set of time-varying data consisting of state *s_t_* (clinical data of the current visit), action *a_t_* (treatment), and reward *r_t_* score (based on clinical outcome). The ultimate goal of RL is to learn a policy *π*, which for any given state, one can select the action that maximizes cumulative future rewards.

The DQN [38] is a type of RL method that has been recently utilized to solve clinical decision problems with continuous state variables [13,39,40]. Referring to the previous DQN work of sepsis treatment in the intensive care unit [13], we applied deep neural networks to calculate the action-value function *Q* that estimates the cumulative rewards for each treatment action at the current visit state. To train the DQN model, two neural networks with the same architecture were used: an evaluation network *Q*(·) and a target network 
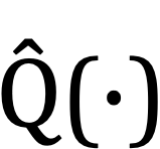
. The evaluation network was used to obtain optimal action max_a_ *Q*(*s_t_, a, θ*) and was trained by the loss function *L*(*Q′, Q*). The target network was used to estimate the expected action-value *Q′* to calculate the loss function *L* and updated its parameters
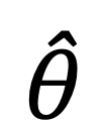
 by slowly tracking the parameters of the evaluation network *θ* every training iteration:
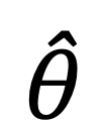
 ←*τ*·*θ*+*θ* (1 – *τ*)·
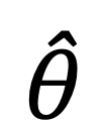
 with update parameter *τ*<1. The loss function *L*(*Q′, Q*) is defined in Equation (1):



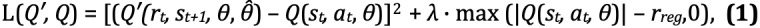



where the expected *Q′* is:



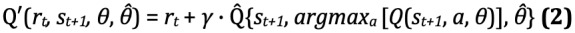



and *r_reg_* is the maximum reward of all *r_t_* that is used in the regularization term to penalize an inexpertly large *Q* value. Here, we used a double-DQN [36] architecture that calculates 
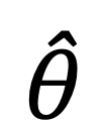
 in Equation (2) through action from the evaluation network *a′*=*argmax_a_* [*Q*(*s_t+1_*, *a*, *θ*)] instead of max
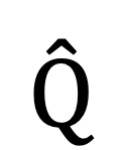
. Double-DQN leads to a more stable learning target and low-variance action-value estimates. Moreover, we used dueling-DQN [37] that adds a dueling architecture in the network to separate the output of the last hidden layer into two streams to learn state values and state-independent action advantages, respectively. We also used a prioritized experience replay [41] method to speed up the training approach. Each training batch was sampled from the training data according to the importance, which was measured by the samples’ temporal-difference error. The complete training procedure of our DQN model can be found in Algorithm S1 in Multimedia Appendix 1.

### Details of DQN Implementation on the Dataset

To present the specific design of the RL-based data-driven model, we take antiglycemic treatment as an example to explain how we trained and applied the DQN in our models.

**Figure 2:**
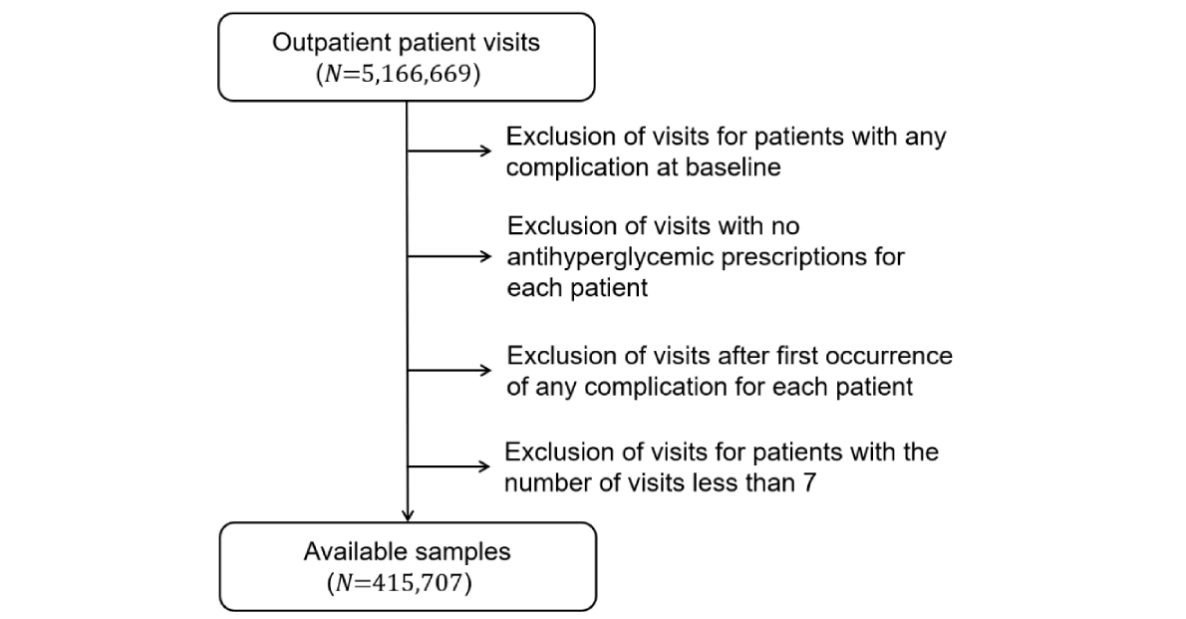
Training set and validation set generation criteria for antiglycemic treatment recommendation.

The given dataset makes up 80% of the entire dataset, which contained 152,527 patients and 5,166,669 outpatient visits. To define outpatient visit samples from the EMRs, we used lab test results (or physical measurements) within a certain time interval before the visit as state variables of its corresponding sample. The complications that occurred before the current outpatient visit were treated as medical history. The criteria used in the generation of a given dataset are shown in Figure 2. Subsequently, the patients in the given dataset were randomly divided into a training set and validation set at a ratio of 8:2.

The features that were selected to define the states for a patient visit included demographics, medical history, disease risks, previous drugs, lab data, and physical measurements. The detailed state information is presented in Table S1 in Multimedia Appendix 1. Among these features, continuous variables were normalized into a common scale, whereas binary variables were represented using 0 or 1. Other categorical variables were converted into multiple binary variables using one-hot encoding. Finally, we obtained a 49-dimension state vector.

For patients with T2DM, antiglycemic medications are usually prescribed based on the currently used drugs [29,32,33]. Thus, to simplify the action space of the DQN, we defined an action of a visit based on medication changes from the previous prescription. The prescription changes included drug changes at the drug class level and dosage changes of some common drugs. In terms of dosage changes, the increase or decrease in dosage of the three most frequently used drugs, namely metformin, basal insulin, and premixed insulin, was considered. Table 1 lists the actions for antiglycemic treatment used in the DQN model. The most common medication adjustment options in the actions were: changing the dosage of a drug, adding an oral antidiabetic drug (OAD), and changing to insulin treatment. Among the action options, “No prescription change” indicates use of the same drugs and dosages as the previous prescription, and “Using xxx insulin” means changing to the specific insulin or insulin combinations.

**Table 1:** Actions of the deep Q network for antiglycemic treatment.

Number	Action
0	No prescription change
1	Increase drug dosage
2	Decrease drug dosage
3	Adding alpha-glucosidase inhibitor
4	Adding dipeptidyl peptidase-4 inhibitor
5	Adding metformin
6	Adding sodium glucose cotransporter-2 inhibitor
7	Adding sulfonylurea
8	Adding thiazolidinedione
9	Adding glucagon-like peptide-1 receptor agonist
10	Using basal insulin
11	Using premix insulin
12	Using basal and prandial insulins

The reward function is usually defined to quantify the effectiveness of the action at each time step. In the antiglycemic DQN, for a patient visit at time *t*, we defined the reward function *r_t_* as shown in Equation (3):

reward_gly_=*a* · sgn(7 – *s_t+1_^HbA_1c_^*) + (–b) · *s_t+1_^Hypo^* + (–c) · *s_t+1_^Final^* · *s_t+1_^CX^***(3)**

where



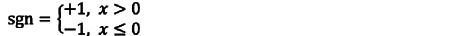
**(4)**


In Equation 3, *s_t_*_+1_*^HbA_1c_^* (%) is the HbA_1c_ of time t+1, *s_t_*_+1_*^Hypo^*, is a binary (1,0) variable representing whether hypoglycemia occurs before time *t*+1, *s_t+1_^Final^* is a binary (1,0) variable representing whether time *t*+1 is the final visit of the patient, and *s_t+1_^CX^* ∈ {–1,1} indicates whether complications or death occur at time *t*+1, where –1 represents “No” and 1 represents “Yes.”

The concept underlying the reward function is to give a positive reward when (1) the HbA_1c_ after 3-6 months reaches the control target (less than 7%), and (2) no complications or death occurred until the last visit of a patient in the next 6 years. A negative reward (ie, penalty) is given when (1) the HbA_1c_ after 3-6 months is not well controlled, (2) a hypoglycemia event occurs in the next 6 months, and (3) a complication or death occurs after the current visit. Based on the importance of these outcomes, we set the coefficients in Equation (1) as follows: *a*=1, *b*=2, *c*=4. For an intermediate visit of a patient, the DQN model is trained to optimize the cumulative reward, which is equal to the current reward plus the next visit’s expected cumulative reward multiplied by a discount factor, *γ*=0.9. Therefore, the DQN model is able to estimate the impact of a current action on both short-term and long-term outcomes.

The network architecture and training settings are provided below. We adopted a fully connected neural network with 2 hidden layers of 64, with 32 units for the Q networks. Each hidden layer contained batch normalization and Leaky-ReLU activation. The input layer was 49 dimensions and the output layer was 14 dimensions, which were the same as the sizes of the state vector and the action space. The learning rate *η* was 0.001, the batch size was 256, and the target network update parameter *τ* was set to 0.01. For regulation, we set the reward threshold *r_reg_*=4 and *λ*=0.5. We trained the DQN model for a maximum of 100,000 iterations using the Adam optimizer [42].

For antihypertensive and lipid-lowering treatments, actions and reward functions of DQNs are shown in Table 2 and Table 3, respectively.

**Table 2:** Actions of the deep Q network for antihypertensive treatment.

Number	Action
0	No drugs
1	Using A^a^
2	Using B^b^
3	Using C^c^
4	Using D^d^
5	Using A and B
6	Using A and C
7	Using A and D
8	Using B and C
9	Using B and D
10	Using C and D
11	Using A, B, and C
12	Using A, B, and D
13	Using A, C, and D
14	Using B, C, and D
15	Using A, B, C, and D

^a^Angiotensin-converting-enzyme inhibitor or angiotensin II receptor blocker.

^b^Beta blocker.

^c^Calcium channel blocker.

^d^Diuretic.

**Table 3:** Actions of the deep Q network for lipid-lowering treatment.

Number	Action
0	No drugs
1	Using statin
2	Using fibrate
3	Using ezetimibe
4	Using statin and fibrate
5	Using statin and ezetimibe
6	Using fibrate and ezetimibe
7	Using statin, fibrate, and ezetimibe

In the antihypertensive DQN, for a patient visit at time *t*, we defined the reward function as in Equation (5):



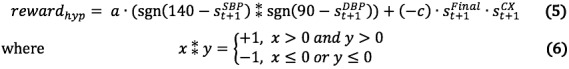



*s_t+1_^SBP^*(mmHg) is the SBP of time *t*+1, *s_t+1_^DBP^* (mmHg) is the DBP of time *t*+1, and other terms are defined as in the reward function of the antiglycemic DQN.

In the lipid-lowering DQN, for a patient visit at time *t*, we defined the reward function as in Equation (7):

*Reward_lip_* = *a* · sgn(2.6 – *s_t+1_^LDL-c^*) + (–*c*) · *s_t+1_^Final^* · *s_t+1_^CX^***(7)**

where *s_t+1_^LDL-c^* (mmol/L) is the LDL-c of time *t*+1, and other terms are defined as in the reward function of the antiglycemic DQN.

### Evaluation Methods

#### Short-Term Outcome Evaluation

Similar to previous works [27,43,44], we took model concordance as the exposure factor, which was determined by whether the actual prescription from the physician is concordant to the model-recommended medication. Thus, we partitioned the patient visits into a model-concordant group and a model-nonconcordant group. The short-term clinical outcomes were then compared between the two groups in terms of the goal-achieving rate of the key parameters, including blood glucose control, blood pressure control, blood lipids control, and hypoglycemia events.

For each short-term outcome, we (1) followed the first 2 exclusion steps in the dataset generation process shown in Figure 2 for the corresponding treatment type (antiglycemic, antihypertensive, or lipid-lowering treatment) and (2) excluded visits without the short-term outcome information. Thus, one patient may contribute different patient-visit samples for evaluation of these outcomes. We applied the corresponding treatment recommendation model onto these patient-visit samples to generate model-recommended medications, and partitioned the patient-visit samples into the model-concordant group and model-nonconcordant group according to the physicians’ prescription. Short-term outcomes were compared between the model-concordant treatment and the model-nonconcordant treatment with significance of differences assessed via a χ^2^ test.

Furthermore, we combined stratification, PS methods, and multivariate regression to adjust confounders. We first stratified the patient visits by the confounder (eg, current HbA_1c_) that was most strongly correlated to the clinical outcome. We then performed the PS inverse probability weighting method [45,46] to adjust multiple confounders for both overall samples and stratified samples, since PS methods have been increasingly used to control confounders [47,48] in observational studies, especially for causal effect analysis. Finally, weighted multivariate logistic regression was applied to adjust for residual imbalances that might exist after PS modeling, and the adjusted odds ratios (ORs) and 95% CIs in multivariate regression were used to reveal the relationship between model concordance and short-term outcome.

#### Long-Term Outcome Evaluation

At the patient level, model-concordant rates were included as independent variables to evaluate the performance of the combined treatments with antiglycemic, antihypertensive, and lipid-lowering medications. The model-concordant rate was calculated for each patient by dividing the number of model-concordant visits by the total number of visits. We defined the model-concordant rate to quantify the extent to which each patient complied with the model recommendations.

We followed a similar process as shown in Figure 2 to generate three test datasets. The difference was that patient visits after the first occurrence time of the corresponding complication were removed instead of the first occurrence time of the earliest occurring complication. To describe the relationship between the patient’s model-concordant rate and the occurrence rate of the long-term outcome, we present illustration curves for each type of treatment and each kind of long-term outcome, and calculated the slopes by fitting the curves with a linear function for exploring the trend. Multivariate logistic regression was further used to investigate the associations between the three types of comprehensive treatments and long-term outcomes, where patients with the three kinds of model-concordant rates were included as test samples. In the multivariate regression, the three concordant rates were included as independent variables, and the predicted risk score at baseline (representing the effects of multiple risk factors on the occurrence of a complication or death) was included as a covariate for confounder adjustment. Coefficients and *P* values are reported for both independent variables and covariates.

## Results

### Patient Characteristics

Of the 36,993 patients in the test data, 18,878 were men (51%). With respect to ethnicity, the majority of the patients were Chinese (69%), followed by Malay (15%) and Indian (11%). By 2019, the median age was 67 years (IQR 59-76) and the median duration of diabetes was 10 years (IQR 6-16).

**Figure 3:**
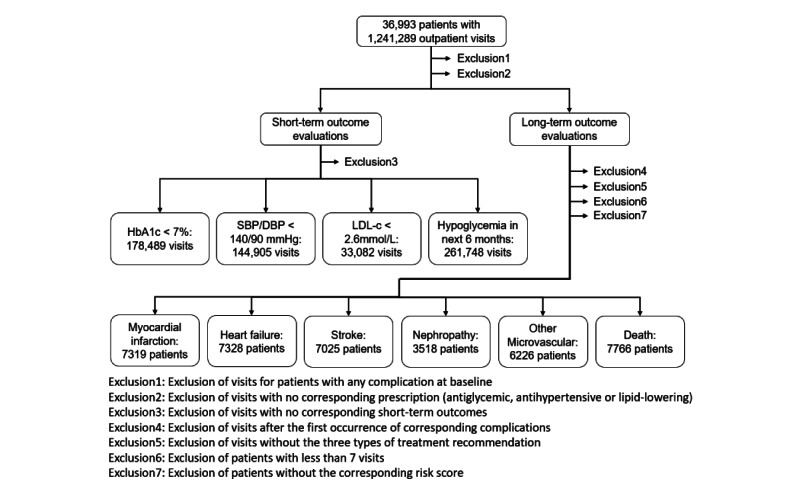
Overview of the exclusion criteria and the number of visits or patients in each evaluation cohort. SBP: systolic blood pressure; DSB: diastolic blood pressure; HbA_1c_: glycated hemoglobin A_1c_; LDL-c: low-density lipoprotein cholesterol.

An overview of the short-term and long-term outcome evaluation cohorts is shown in Figure 3. Further information is provided in the following two subsections.

### Short-Term Outcomes at the Patient-Visit Level

To evaluate the short-term outcomes, we took model concordance as the exposure variable to evaluate the effect of our treatment recommendation model at the patient-visit level. We generated the test datasets separately for different short-term outcomes, namely the percentages of patient visits with well-controlled parameters (HbA_1c_<7% [53 mmol/mol], SBP/DBP<140/90 mmHg, LDL-c<2.6 mmol/L [100 mg/dl]) after 3-6 months of therapy. For a hypoglycemia event, the occurrence rate in the following 6 months was compared between two groups. In short-term evaluation, potential confounding factors were adjusted by stratification, the PS weighting method, and multivariate regression, such as age, gender, and ethnicity.

Specifically, the model concordance was defined at the level of the standard drug class, since the treatment recommendation models output the standard drug class rather than the specific brand name. For example, if the model recommends alpha-glucosidase inhibitors (AGI), the patient visit with a prescription for Acarbose (a type of drug belonging to the drug class AGI) is model-concordant. Only the top-ranking recommended medication for antiglycemic, antihypertensive, and lipid-lowering therapy was considered for evaluation.

After meeting all exclusion criteria, a total of 178,489 visits were included to evaluate the short-term clinical outcomes of HbA_1c_ control. Of the total samples, 78,670 patient visits (44.08%) were model-concordant and 99,819 (55.92%) were nonconcordant. The characteristics of the model-nonconcordant and model-concordant groups in the test data are shown in Table 4 for short-term blood glucose control evaluation, and patient characteristics for other short-term outcomes (eg, blood pressure control) are shown in Tables S2-S4 in Multimedia Appendix 1.

**Table 4:** Characteristics of the glycated hemoglobin A_1c_ (HbA_1c_) cohort.

Variables		Model-nonconcordant group (n=99,819)	Model-concordant group (n=78,670)	*P* value^a^
Age (years), mean (SD)		64.17 (12.23)	64.61 (11.68)	<.001
Gender (female), n (%)		48,450 (48.5)	39,386 (50.1)	<.001
**Ethnicity, n (%)**				
	Chinese	69,019 (69.1)	56,622 (72.0)	<.001
	Indian	10,658 (10.7)	7,416 (9.4)	<.001
	Malay	15,961 (16.0)	11,701 (14.9)	<.001
Smoker/exsmoker, n (%)		10,008 (10.0)	7644 (9.7)	.03
Duration of diabetes (years), mean (SD)		12.14 (8.11)	10.01 (7.52)	<.001
HbA_1c_ (%), mean (SD)		8.08 (1.39)	6.94 (1.15)	<.001
SBP^b^ (mmHg), mean (SD)		133.06 (16.77)	131.71 (16.47)	<.001
DBP^c^ (mmHg), mean (SD)		69.72 (9.40)	69.33 (9.35)	<.001
LDL-c^d^ (mmol/L) mean (SD)		2.27 (0.76)	2.19 (0.70)	<.001
TG^e^ (mmol/L), mean (SD)		1.61 (0.95)	1.46 (0.79)	<.001
BMI (kg/m^2^), mean (SD)		26.75 (5.79)	26.54 (5.95)	<.001
eGFR^f^ (mL·min^-1^·1.73m^–2^), mean (SD)		80.56 (33.84)	84.14 (30.65)	<.001
Hypertension, n (%)		92,235 (92.4)	72,066 (91.6)	<.001
Hypercholesterolemia, n (%)		97,506 (97.7)	76,385 (97.1)	<.001
Macrovascular complications, n (%)		26,347 (26.4)	19,604 (24.9)	<.001
Microvascular complications, n (%)		44,904 (45.0)	29,614 (37.6)	<.001

^a^Based on a *t* test or χ^2^ test.

^b^SBP: systolic blood pressure.

^c^DBP: diastolic blood pressure.

^d^LDL-c: low-density lipoprotein cholesterol.

^e^TG: triglycerides.

^f^eGFR: estimated glomerular ﬁltration rate.

The evaluation results of short-term outcomes, including HbA_1c_, SBP/DBP, LDL-c control, and hypoglycemia event, were based on test samples with corresponding outcome data during the follow-up period. After confounder adjustment for patient characteristics, the model-concordant treatments were associated with good blood glucose control, good blood pressure control, and good blood lipid control compared with model-nonconcordant treatments. There was no significant difference in the occurrences of hypoglycemia events between model-concordant treatments and model-nonconcordant treatments (Table 5).

We further stratified the patient visits by the confounder that was most strongly correlated to the clinical outcome. For the outcome of glucose control (ie, HbA_1c_ after 3-6 months), the current HbA_1c_ of the patient was used to stratify the patient visits into three groups of low (<7%), medium (7-9%), and high (>9%) levels. The short-term evaluation was performed on each group separately (Table 6), showing that model-concordant treatments were associated with improved short-term HbA_1c_ outcomes (ie, higher HbA_1c_ goal-achieving rate) in each group.

**Table 5:** Short-term clinical outcomes in the model-concordant and model-nonconcordant groups.

Short-term outcomes			Samples, n		Before adjustment				After propensity score weighting adjustment				
					Incidence, n (%)		*P* value		OR^a^		95% CI		*P* value
**Antiglycemic treatment^b^ (HbA_1c_)^c^ <7%)**							<.001		1.73		1.69-1.76		<.001
	Concordant	78,670		48,263 (61.35)									
	Nonconcordant	99,819		21,507 (21.55)									
**Antihypertensive treatment^d^ (SBP^e^/DBP^f^<140/90 mmHg)**							<.001		1.26		1.23-1.29		<.001
	Concordant	80,868		62,058 (76.74)									
	Nonconcordant	64,037		35,327 (55.17)									
**Lipid-lowering treatment^g^ (LDL-c^h^<2.6 mmol/L)**							<.001		1.28		1.22-1.35		<.001
	Concordant	14,985		10,702 (71.42)									
	Nonconcordant	18,097		10,028 (55.41)									
**Antiglycemic treatment^i^ (Hypoglycemia in next 6 months)**							<.001		0.97		0.91-1.02		0.22
	Concordant	113,343		1497 (1.32)									
	Nonconcordant	148,405		3009 (2.03)									

^a^OR: odds ratio.

^b^Confounders considered: age, gender, ethnicity, smoking, duration of diabetes, HbA_1c_, SBP/DBP, LDL-c, triglycerides (TG), BMI, estimated glomerular filtration rate (eGFR), hypertension, hypercholesterolemia, macrovascular complication, microvascular complication, hypoglycemia history.

^c^HbA_1c_: glycated hemoglobin A_1c_.

^d^Confounders considered: age, gender, ethnicity, smoking, duration of diabetes, HbA_1c_, SBP/DBP, LDL-c, TG, BMI, eGFR, hypercholesterolemia, myocardial infarction, unstable angina, heart failure, stroke, nephropathy.

^e^SBP: systolic blood pressure.

^f^DBP: diastolic blood pressure.

^g^Confounders considered: age, gender, ethnicity, smoking, duration of diabetes, HbA_1c_, SBP/DBP, LDL-c, TG, BMI, eGFR, alanine transaminase, macrovascular complication, nephropathy.

^h^LDL-c: low-density lipoprotein cholesterol.

^i^Confounders considered: age, gender, ethnicity, smoking, duration of diabetes, HbA_1c_, SBP, LDL-c, BMI, serum creatinine, hypertension, atrial fibrillation, macrovascular complication, microvascular complication.

**Table 6:** Short-term outcome for antiglycemic treatment based on current glycated hemoglobin A_1c_ (HbA_1c_)

Current HbA_1c_ level		Samples, n	Before adjustment		After propensity score weighting adjustment		
			Incidence, n (%)	*P* value	OR^b^	95% CI	*P* value
**Low (<7%)**				<.001	1.79	1.69-1.89	<.001
	Concordant	60,428	43,836 (72.54)				
	Nonconcordant	5959	4034 (67.7)				
**Medium (7-9%)**				<.001	1.76	1.69-1.83	<.001
	Concordant	14,025	3816 (27.21)				
	Nonconcordant	76,423	16,365 (21.41)				
**High (>9%)**				<.001	1.83	1.63-2.05	<.001
	Concordant	4217	611 (14.49)				
	Nonconcordant	17,437	1108 (6.35)				

^a^Confounders considered: age, gender, ethnicity, smoking, duration of diabetes, HbA_1c_, systolic blood pressure/diastolic blood pressure, low-density lipoprotein cholesterol, triglycerides, BMI, estimated glomerular filtration rate, hypertension, hypercholesterolemia, macrovascular complication, microvascular complication, hypoglycemia history.

^b^OR: odds ratio.

### Long-Term Outcomes at the Patient Level

**Figure 4:**
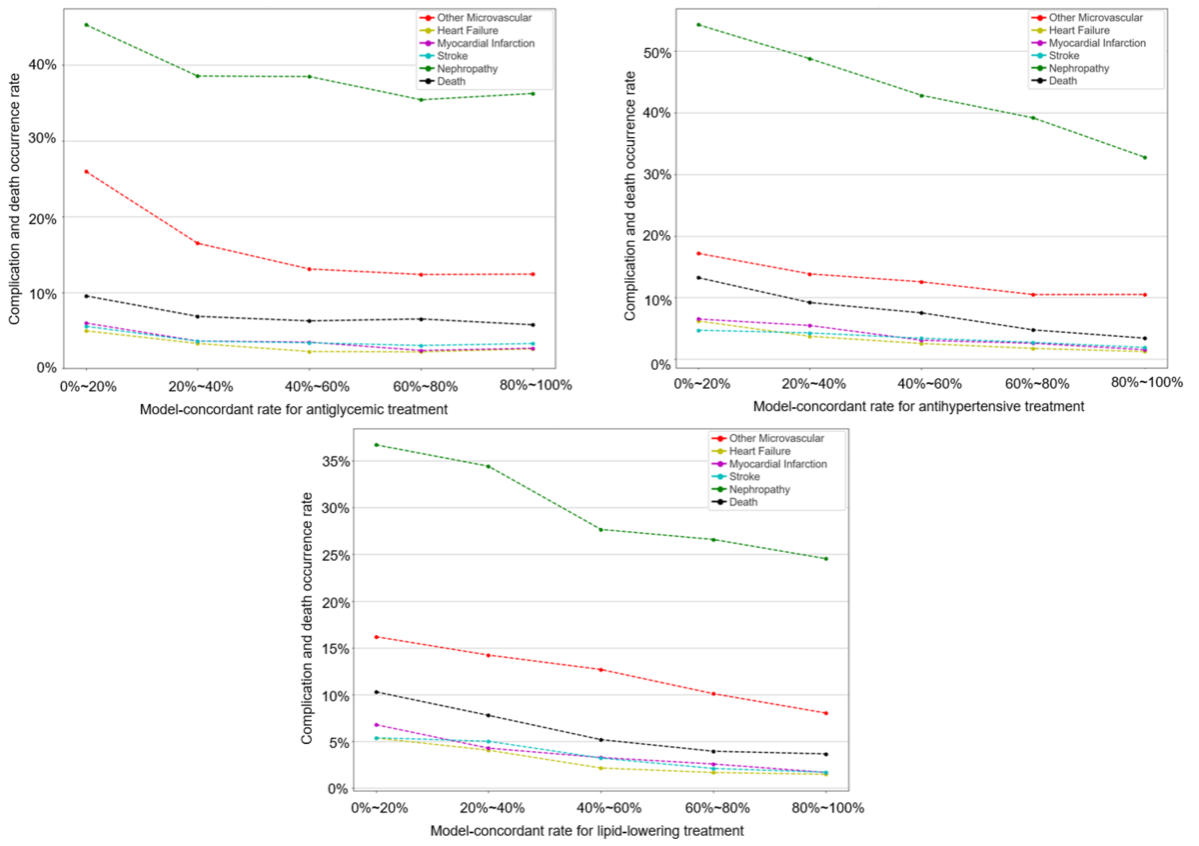
Relationship between patient’s model-concordant rate and the occurrence rate of long-term outcomes for all patients with respect to antiglycemic, antihypertensive, and lipid-lowering treatment, respectively.

Figure 4 illustrates the relationship between the patient’s model-concordant rate and the occurrence rate of long-term clinical outcomes for all patients with respect to antiglycemic, antihypertensive, and lipid-lowering therapy. Specifically, the patients were divided into different groups according to the patient’s model-concordant rate (eg, every 20% as a group), and the occurrence rate of complications or death in each group was computed. In general, the curves show a downward trend. In other words, there is a negative correlation between the model-concordant rate and the occurrence rate of complications or death; the higher the patient’s model-concordant rate, the lower the occurrence rate of complications or death. Table 7 shows the slope of each curve by fitting to a straight line using all data points, which indicates the extent of the downward trend. In addition, the number of patients in each long-term outcome evaluation cohort is shown in Table S5 in Multimedia Appendix 1.

Furthermore, for combined treatments with antiglycemic, antihypertensive, and lipid-lowering drugs, we evaluated if the patient’s model-concordant rate for the three types of treatments had a positive impact on the reduction of the complication or death risk by multivariate regression. Only patients with all three model-concordant rates of antiglycemic, antihypertensive, and lipid-lowering treatment were included in the multivariate regression model. Table 8 shows the multivariate regression results of each long-term outcome with confounder adjustment for the corresponding risk score generated by the prediction model. All of the prediction models, based on XGBoost, outperformed the clinical baseline models [49-53] and demonstrated good prediction capability, with an area under the receiver operating characteristic curve ranging from .711 to .874. The model-concordant rate for antiglycemic treatment had a negative correlation with the occurrence of major complications and death, with coefficients ranging from –1.12 to –.33. A similar result was found for the model-concordant rate for antihypertensive treatment (coefficient range –1.44 to –.40) and the model-concordant rate for lipid-lowering treatment (coefficient range –1.17 to –.52). All of these coefficients were significant (*P*<.05), except for the coefficients of antiglycemic treatment and antihypertensive treatment in the evaluation of stroke outcome. This implies that the patients whose treatments were more concordant with the model recommendation were more likely to be associated with a lower risk of diabetes complications (including both macrovascular and microvascular complications) and death. All of the coefficients for the risk score had positive values, which also validated the soundness of the risk prediction models. In addition, the number of patients with or without the corresponding long-term outcomes in multivariate regression are shown in Table S6 in Multimedia Appendix 1.

**Table 7:** Slopes of patient-level long-term outcome evaluation curves in Figure 4.

Curves fit	Antiglycemic treatment	Antihypertensive treatment	Lipid-lowering treatment
Myocardial infarction	–0.0384	–0.0617	–0.0581
Heart failure	–0.0312	–0.057	–0.0502
Stroke	–0.0261	–0.0389	–0.0535
Nephropathy	–0.1098	–0.2601	–0.1670
Other microvascular	–0.1584	–0.0827	–0.1007
Death	–0.0419	–0.1240	–0.0881

**Table 8:** Multivariate regression results for long-term outcomes.

Long-term outcome	Antiglycemic treatment model concordance rate		Antihypertensive treatment model concordance rate			Lipid-lowering treatment model concordance rate			Risk score (%)	
	Coefficient (β)	*P* value	Coefficient (β)	*P* value	Coefficient (β)		*P* value	Coefficient (β)		*P* value
Myocardial infarction	–1.1150	<.001	–.8018	<.001	–1.0065		<.001	.0998		<.001
Heart failure	–.6294	.04	–1.4414	<.001	–1.0416		<.001	.0653		<.001
Stroke	–.3288	.24	–.4871	.06	–1.1715		<.001	.1210		<.001
Nephropathy	–.5667	<.001	–1.2648	<.001	–.5182		<.001	.0072		<.001
Other Microvascular	–.5382	.001	–.3956	.004	–.6296		<.001	.0552		<.001
Death	–.4922	.02	–.8872	<.001	–.9835		<.001	.0527		<.001

### Medication Pattern

In all patient visits, the percentages of model-concordant visits were 43.30%, 51.25%, and 58.93% for antiglycemic, antihypertensive, and lipid-lowering therapy, respectively. To analyze the distribution characteristics for model concordance, we compared the antiglycemic medication patterns between physicians’ prescriptions and model recommendations.

**Figure 5:**
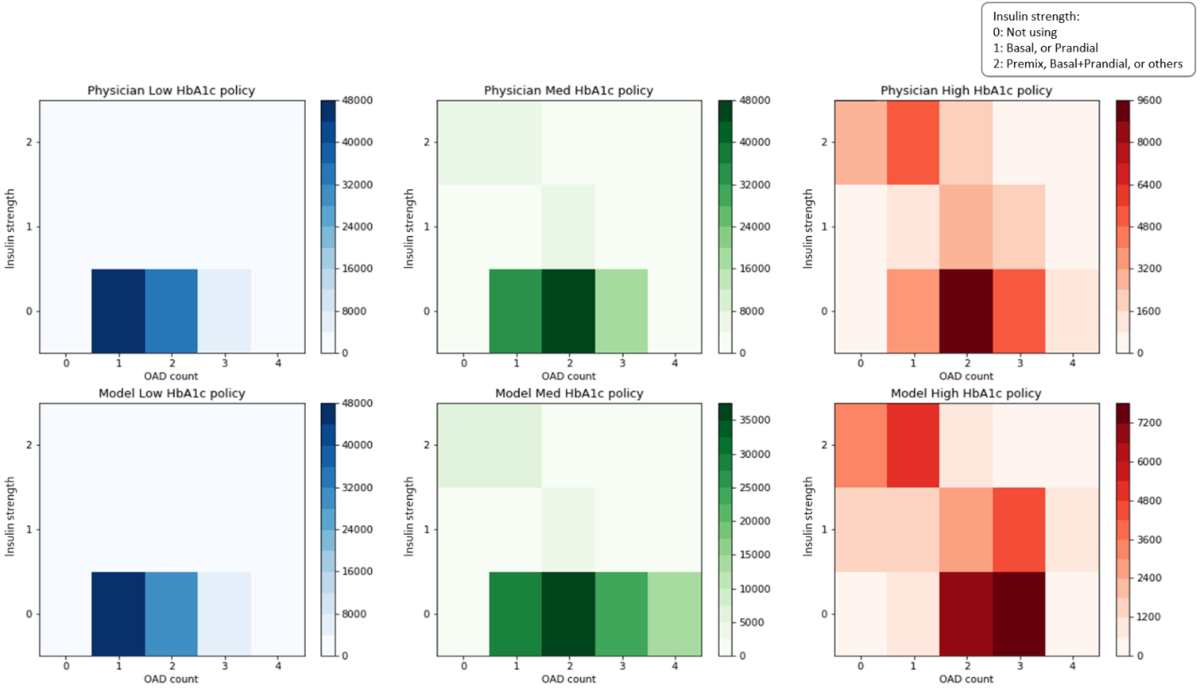
Medication pattern comparison between physicians’ prescriptions and model recommendations. HbA_1c_: glycated hemoglobin A_1c_; OAD: oral antidiabetic drug.

Figure 5 depicts the medication patterns used by physicians (top panels) and the model (bottom panels) for the three different groups. All patient-visit samples were categorized into three groups based on the current HbA_1c_ as follows: low (<7%), medium (7-9%), and high (>9%). For each group, the medication patterns of physicians’ prescriptions and model recommendations are visualized by 2D histograms, in which the x-axis represents the number of OADs and the y-axis represents the intensity of insulin (a value of 0 indicates no insulin used, 1 indicates single use of basal insulin or prandial insulin, and 2 indicates a premix or combination use of basal and prandial insulin, or others). The color indicates the usage number of corresponding medication patterns. First, Figure 5 shows that medication patterns of model recommendations are consistent with clinical knowledge, as most patients in the low group were prescribed with a single OAD, whereas patients in the medium and high groups showed increased use of multi-OAD, insulin, and insulin plus OAD. Second, the medication patterns of model recommendations are visually similar to those of physicians’ prescriptions in the low group, whereas in the medium and high groups, the patterns of model recommendations are more vigorous than those of physicians’ prescriptions. This indicates that the model learns from the data, showing that active adjustment of the medication for the patients in medium and high groups may be associated with a better clinical outcome.

## Discussion

In this work, we built our treatment recommendation model based on 80% of the data in SingHealth Diabetes Registry and evaluated its effectiveness with the remaining 20% of data. The strengths of this retrospective study are two-fold. First, the diabetes registry used for model building and evaluation is of good quality. It consists of the medical records for a large patient population, covers a long-term span of 6 years, and includes different types of diabetes complications (macrovascular and microvascular). Second, the methods used for the evaluation are comprehensive. For the treatment recommendation, we considered the concordance of three types of treatments (antiglycemic, antihypertensive, and lipid-lowering treatment) and evaluated two types of outcomes, namely the control of key indicators in the short term and the occurrences of diabetes complications in the long term.

The treatments are recommended by a combination of a knowledge-driven model and a data-driven model. For the knowledge-driven model, we incorporated renal dosing and contraindications for specific medications so as to align with standards of care. For example, the model will not recommend an increase in the metformin dose in subjects with an estimated glomerular filtration rate (eGFR) <45 mL·min^-1^·1.73 m^–2^ and will recommend the discontinuation of metformin if the eGFR is below 30 mL·min^-1^·1.73 m^–2^ [29]. For the data-driven model, we ranked the candidates by the expected clinical outcomes.

In our study, the antiglycemic medications recommended by our model were the same as the actual prescriptions of the physicians in 43.30% of patient visits. The percentages of model-concordant visits for antihypertensive medications and lipid-lowering medications were 51.25% and 58.93%, respectively. For the treatment recommendation evaluation, patients with more model-concordant treatments had better control of blood glucose (OR 1.73, 95% CI 1.69-1.76), blood pressure (OR 1.26, 95% CI 1.23-1.29), and blood lipids (OR 1.28, 95% CI 1.22-1.35), as well as a lower risk of diabetes complications (coefficients of regression ranging from –1.44 to –.33). In addition, there was no significant difference (OR 0.97, 95% CI 0.91-1.02) on the risk of hypoglycemia events between model-concordant treatments and model-nonconcordant treatments. These evaluation results suggested that the treatment recommendation model has good potential to guide physicians in prescribing medications that could help to achieve better clinical outcomes.

Our study has some limitations. First, the algorithm was more aggressive in recommending complex treatment regimens than the actual physicians’ prescriptions, especially in the medium and high HbA_1c_ groups. The dataset is built based on the EMRs of patients with T2DM. Some information that can influence the choice of a physician’s prescription may be missing in the data. For example, a physician may default to repeating a previous prescription because of the patient’s reluctance to change medications. Such patient preference will not be recorded in the data and hence not used in the treatment recommendation model. This is reflective of “human bias” for less complex treatment regimens in real-world clinical practice. Second, selection bias may exist in this study. For example, when evaluating the long-term clinical outcome, we selected the patients with a number of visits greater than a threshold. As such, newly added patients in 2018 were hardly selected. Third, the unified therapeutic targets were used in this study without considering personalized control targets for individual patients. For example, the control goal of blood glucose was set to HbA_1c_<7% in this study. However, for elderly patients and patients with recurrent hypoglycemia, the HbA_1c_ goal could be less strict. Fourth, the hypoglycemia episodes that did not end up requiring admissions may be reported by the patients but are seldom coded in the EMRs as a blood test or diagnosis that the algorithm can identify [54]. Thus, our analysis only considered severe hypoglycemia events with hospital admissions. Finally, although we performed the PS weighting method and multivariate regression analysis to control for differences in demographic and clinical conditions when evaluating the association between model concordance and clinical outcomes, a conclusion regarding the causal effect of model concordance cannot be made based on the observed association due to the limitations of a retrospective study.

In future work, the treatment recommendation model can be further evaluated in a prospective study by piloting an interactive treatment recommendation system in a real-world clinical practice. Finally, the knowledge-driven and data-driven models need to be optimized regularly to make use of newly collected EMR data, and to incorporate the latest clinical guidelines and new classes of drugs.

## Appendix

Multimedia Appendix 1 Supplementary data: Algorithm S1, Tables S1-S6.

## Abbreviations

AGI: alpha-glucosidase inhibitors
DBP: diastolic blood pressure
DQN: deep Q network
eGFR: estimated glomerular filtration rate
EMR: electronic medical record
HbA_1c_: glycated hemoglobin A_1c_
LDL-c: low-density lipoprotein cholesterol
OAD: oral anti-diabetic drug
OR: odds ratio
PS: propensity score
RCT: randomized controlled trial
RL: reinforcement learning
SBP: systolic blood pressure
SingHealth: Singapore Health Services
T2DM: type 2 diabetes mellitus
