# Supplementary material for: Effective Treatment Recommendations for Type 2 Diabetes Management Using Reinforcement Learning: Treatment Recommendation Model Development and Validation
Source: J Med Internet Res. 2021 Jul 22;23(7):e27858. doi: 10.2196/27858 (PMC8367185; doi:10.2196/27858)
Supplement: Multimedia Appendix 1 [file jmir_v23i7e27858_app1.docx]

# Supplementary Data for

# Effective Treatment Recommendations for Type 2 Diabetes Management Using Reinforcement Learning: Treatment Recommendation Model Development and Validation

**Supplementary Algorithm S1.** DQN training procedure.

| **Algorithm S1 DQN training procedure** |
| --- |
| 1. Calculate rewards for each state-action pair and generate samples of transition $T_{j}=(s_{t},a_{t},s_{t+1},r_{j})$ to form the experience replay memory $\Pi$ of size $N$. 2. Initialize the sampling probability $p_{j}=r_{j}$ and importance-sampling weight $w_{j}=\left( N\cdot p_{j} \right)^{-\beta}/\max_{i}(w_{i})$ 3. Initialize variables of evaluation network $\theta$ and target network $\hat{\theta}$ 4. **for** each iteration step **do** 5. Sample a batch of transitions from $\Pi$ by $p_{j}$ 6. **for** each sample in the batch **do** 7. Select next state optimal action $a_{t+1}^{'}=\arg\max_{a}\left( Q\left( s_{t+1}, a,\theta\right) \right)$ for each transition 8. Compute expected action-value $Q'$ using Equation (2) by target network 9. Compute action value of current action $Q\left( s_{t}, a_{t},\theta\right)$ by evaluation network 10. Compute Q-loss $L$ using Equation (1) 11. **end for** 12. Compute weighted Q-loss of the batch $L\left( j \right)= \sum_{batch} w_{j}\cdot L_{j}$ 13. Update $\theta$ by training the network via $L\left( j \right)$ 14. Update $\hat{\theta}=\tau\cdot\theta+(1-\tau)\cdot\hat{\theta}$ 15. Update $p_{j}={(\left\vert Q^{'}-Q \right\vert+\varepsilon)}^{\alpha}$ and $w_{j}=\left( N\cdot p_{j} \right)^{-\beta}/\max_{i}(w_{i})$ 16. **end for** |

**Supplementary Table S1.** The State features of DQN for anti-glycemic treatment recommendation.

| **Class of feature** | **Feature** | **Class of feature** | **Feature** |
| --- | --- | --- | --- |
| Demographic information | Gender | Physical measurement | Body mass index |
|  | Age |  | Systolic blood pressure |
|  | Ethnicity |  | Diastolic blood pressure |
|  | Living condition | Lab test | Glycated hemoglobin |
|  | Smoking history |  | Fasting plasma glucose |
| Medical history | Diabetes duration |  | Serum creatinine |
|  | Hypertension |  | High-density lipoprotein cholesterol |
|  | Hypercholesterolemia |  | Low-density lipoprotein cholesterol |
|  | Hypoglycemia |  | Total cholesterol |
| Risk of complication or death | Myocardial Infarction |  | Triglycerides |
|  | Heart Failure |  | Alanine aminotransferase |
|  | Stroke |  | Serum urea |
|  | Nephropathy | Previous drug usage | Previous prescription time |
|  | Diabetic eye/foot/neuropathy |  | Oral antidiabetic drugs |
|  | Severe Hypoglycemia |  | Insulins |
|  | Death |  | Drug dosages |

Supplementary Tables S2-S4 are patient characteristics for short-term outcomes (blood pressure control, blood lipids control, and hypoglycemia event). Continuous data are presented as the mean (SD) and categorical data are presented as n (%). Supplementary Table S5 shows the number of test samples at the patient level for each type of treatment and each kind of complication or death.

**Supplementary Table S2.** The characteristics of SBP/DBP cohort.

| **Variables** | **Model-non-concordant group (n=64,037)** | **Model-concordant group (n=80,868)** | **P-value** |
| --- | --- | --- | --- |
| Age (years), mean (SD) | 68.83 (10.99) | 66.17 (10.41) | <.001 |
| Gender (Female), n (%) | 31825 (49.7) | 40544 (50.1) | .10 |
| Ethnicity (Chinese), n (%) | 45,782 (71.5) | 59,655 (73.8) | <.001 |
| Ethnicity (Indian), n (%) | 5,610 (8.8) | 7,553 (9.3) | <.001 |
| Ethnicity (Malay), n (%) | 10,222 (16.0) | 11,004 (13.6) | <.001 |
| Smoker/ex-smoker, n (%) | 5,964 (9.3) | 7,511 (9.3) | .88 |
| Duration of diabetes (years), mean (SD) | 11.73 (8.47) | 10.62 (7.62) | <.001 |
| HbA1c (%), mean (SD) | 7.38 (1.44) | 7.29 (1.32) | <.001 |
| SBP (mmHg), mean (SD) | 144.09 (17.79) | 125.90 (10.66) | <.001 |
| DBP (mmHg), mean (SD) | 72.13 (10.35) | 67.42 (8.26) | <.001 |
| LDL-c (mmol/L), mean (SD) | 2.25 (0.75) | 2.17 (0.67) | <.001 |
| TG (mmol/L), mean (SD) | 1.61 (0.96) | 1.49 (0.76) | <.001 |
| BMI (kg/m^2^), mean (SD) | 26.73 (5.81) | 26.64 (5.65) | .002 |
| eGFR (mL·min^-1^·1.73m^-2^), mean (SD) | 69.85 (30.04) | 84.55 (29.20) | <.001 |
| Hypertension, n (%) | 62,738 (98.0) | 78,900 (97.6) | <.001 |
| Myocardial infarction, n (%) | 2,763 (4.3) | 2,106 (2.6) | <.001 |
| Unstable angina, n (%) | 1,117 (1.7) | 1,110 (1.4) | <.001 |
| Heart failure, n (%) | 3,143 (4.9) | 2,106 (2.6) | <.001 |
| Stroke, n (%) | 6,164 (9.6) | 5,687 (7.0) | <.001 |
| Nephropathy, n (%) | 38,083 (59.5) | 21,959 (27.2) | <.001 |

**Supplementary Table S3.** The characteristics of LDL cohort.

| **Variables** | **Model-non-concordant group (n=18,097)** | **Model-concordant group (n=14,985)** | **P-value** |
| --- | --- | --- | --- |
| Age (years), mean (SD) | 63.90 (11.10) | 64.06 (11.65) | .19 |
| Gender (Female), n (%) | 9,917 (54.8) | 8,283 (55.3) | .39 |
| Ethnicity (Chinese), n (%) | 11,309 (62.5) | 10,057 (67.1) | <.001 |
| Ethnicity (Indian), n (%) | 2,282 (12.6) | 1,725 (11.5) | .002 |
| Ethnicity (Malay), n (%) | 3,357 (18.6) | 2,457 (16.4) | <.001 |
| Smoker/ex-smoker, n (%) | 2,597 (14.4) | 1,861 (12.4) | <.001 |
| Duration of diabetes (years), mean (SD) | 10.57 (8.22) | 10.37 (8.52) | .09 |
| HbA1c (%), mean (SD) | 7.71 (1.72) | 7.43 (1.54) | <.001 |
| SBP (mmHg), mean (SD) | 135.39 (19.91) | 133.95 (18.85) | <.001 |
| DBP (mmHg), mean (SD) | 70.76 (10.39) | 70.30 (10.22) | .001 |
| LDL-c (mmol/L), mean (SD) | 2.91 (0.93) | 2.37 (1.01) | <.001 |
| TG (mmol/L), mean (SD) | 1.83 (0.88) | 1.55 (0.79) | <.001 |
| BMI (kg/m^2^), mean (SD) | 26.90 (5.56) | 26.88 (7.04) | .72 |
| eGFR (mL·min^-1^·1.73m^-2^), mean (SD) | 72.44 (32.55) | 75.75 (32.72) | <.001 |
| ALT (U/L), mean (SD) | 25.05 (16.39 | 25.30 (15.85) | .18 |
| Macrovascular complications, n (%) | 9,601 (53.1) | 5,085 (33.9) | <.001 |
| Nephropathy, n (%) | 9,424 (52.1) | 7,141 (47.7) | <.001 |

**Supplementary Table S4.** The characteristics of Hypoglycemia cohort.

| **Variables** | | **Model-non-concordant group (n=148,405)** | **Model-concordant group (n=113,343)** | **P-value** |
| --- | --- | --- | --- | --- |
| Age (years), mean (SD) | 63.79 (12.51 | 64.41 (11.94) | <.001 |  |
| Gender (Female), n (%) | 72,938 (49.1) | 57,490 (50.7) | <.001 |  |
| Ethnicity (Chinese), n (%) | 100,740 (67.9) | 80,674 (71.2) | <.001 |  |
| Ethnicity (Indian), n (%) | 16,726 (11.3) | 11,091 (9.8) | <.001 |  |
| Ethnicity (Malay), n (%) | 24,500 (16.5) | 17,134 (15.1) | <.001 |  |
| Smoker/ex-smoker, n (%) | 15,654 (10.5) | 11,431 (10.1) | <.001 |  |
| Duration of diabetes (years), mean (SD) | 12.06 (8.19) | 9.97 (7.62) | <.001 |  |
| HbA1c (%), mean (SD) | 8.16 (1.50) | 6.98 (1.24) | <.001 |  |
| SBP (mmHg), mean (SD) | 133.35 (17.27) | 132.13 (16.82) | <.001 |  |
| DBP (mmHg), mean (SD) | 2.29(0.80) | 2.21 (0.73) | <.001 |  |
| LDL-c (mmol/L), mean (SD) | 26.77 (5.75) | 26.56 (6.11) | <.001 |  |
| SCR (umol/L), mean (SD) | 93.81 (73.27) | 84.50 (61.48) | <.001 |  |
| Hypertension, n (%) | 136,128 (91.7) | 103,092 (91.0) | <.001 |  |
| Atrial fibrillation, n (%) | 5,498 (3.7) | 4,183 (3.7) | .86 |  |
| Macrovascular complications, n (%) | 40,989 (27.6) | 29,567 (26.1) | <.001 |  |
| Microvascular complications, n (%) | 70,080 (47.2) | 44,984 (39.7) | <.001 |  |

**Supplementary Table S5**. The number of patients in each long-term outcome evaluation cohort.

| **Long-term outcome** | **Anti-glycemic curves** | **Anti-hypertensive curves** | **Lipid-lowering curves** | **Multivariate analysis** |
| --- | --- | --- | --- | --- |
| Myocardial infarction | 13,718 | 13,516 | 10,369 | 7,319 |
| Heart failure | 13,681 | 13,382 | 10,372 | 7,328 |
| Stroke | 13,206 | 12,892 | 10,012 | 7,025 |
| Nephropathy | 9,157 | 8,099 | 5,441 | 3,518 |
| Other microvascular complications | 12,527 | 12,146 | 9,146 | 6,226 |
| Death | 14,205 | 14,117 | 10,994 | 7,766 |

**Supplementary Table S6.** The number and rate of patients with bad outcome in the multivariate analysis.

| **Long-term outcome** | **Number of patients in the cohort** | **Number of patients with bad outcome** | **Rate of patients with bad outcome** |
| --- | --- | --- | --- |
| Myocardial infarction | 7,319 | 227 | 3.1% |
| Heart failure | 7,328 | 167 | 2.3% |
| Stroke | 7,025 | 189 | 2.7% |
| Nephropathy | 3,518 | 1,143 | 32.5% |
| Other Microvascular | 6,226 | 689 | 11.1% |
| Death | 7,766 | 375 | 4.8% |
